# Composite Fiber Networks Based on Polycaprolactone and Bioactive Glass-Ceramics for Tissue Engineering Applications

**DOI:** 10.3390/polym12081806

**Published:** 2020-08-12

**Authors:** Sorin-Ion Jinga, Claudiu-Constantin Costea, Andreea-Ioana Zamfirescu, Adela Banciu, Daniel-Dumitru Banciu, Cristina Busuioc

**Affiliations:** 1 Faculty of Applied Chemistry and Materials Science, University POLITEHNICA of Bucharest, RO-011061 Bucharest, Romania; sorinionjinga@yahoo.com; 2 Faculty of Medical Engineering, University POLITEHNICA of Bucharest, RO-011061 Bucharest, Romania; claudiuclau653@yahoo.com (C.-C.C.); zamfirescu.andreea96@gmail.com (A.-I.Z.); adela.banciu79@gmail.com (A.B.); danieldumitrubanciu@gmail.com (D.-D.B.)

**Keywords:** polycaprolactone, glass-ceramics, composites, electrospinning, bone regeneration

## Abstract

In this work, composite fibers connected in three-dimensional porous scaffolds were fabricated by electrospinning, starting from polycaprolactone and inorganic powders synthesized by the sol-gel method. The aim was to obtain materials dedicated to the field of bone regeneration, with controllable properties of bioresorbability and bioactivity. The employed powders were nanometric and of a glass-ceramic type, a fact that constitutes the premise of a potential attachment to living tissue in the physiological environment. The morphological characterization performed on the composite materials validated both the fibrous character and oxide powder distribution within the polymer matrix. Regarding the biological evaluation, the period of immersion in simulated body fluid led to the initiation of polymer degradation and a slight mineralization of the embedded particles, while the osteoblast cells cultured in the presence of these scaffolds revealed a spatial distribution at different depths and a primary networking tendency, based on the composites’ geometrical and dimensional features.

## 1. Introduction

Since the future belongs to biomimetic and multifunctional materials, as well as to the identification of the right proportions between multiple components in order to achieve synergistic contributions, the solution to the current challenges in tissue engineering might be related to the strategy of combining bioactive glass or glass-ceramic nanoparticles with a bioresorbable polymeric matrix and possibly other active phases [1]. In this way, by carefully designing the interfaces between individual components and meticulously finding the optimal processing parameters, cell adhesion and proliferation, osteogenic and angiogenic properties, and maybe even additional functionalities could be improved to a significant extent [2].

Starting from the premise that a functional system dedicated to bone regeneration must exhibit a suitable mechanical resistance, tailored degradability and induced mineralization, so as to address large defects and withstand relevant loads, a combination of several organic and inorganic phases seems to be an option that overcomes the majority of problems formulated in clinical practice [3,4]. More than that, the stimulation of cellularization and control of vascularization represent the top priorities for a successful implementation of new models of substitutes [1].

Thus, engineered scaffolds based on biodegradable polymers and amorphous or partially crystallized silicate systems offer extensive perspectives due to the advantages brought by each constituent. In this context, polycaprolactone (PCL) is a biocompatible and biodegradable polyester that has been frequently used in the last decades due its easy availability, cost efficacy and suitability for modification; due to its physiochemical, biological and mechanical properties, it lends itself to being used as biomaterial for bone scaffolds [5]. As a fabrication technique, electrospinning has lately gained popularity due to the capability of providing tunable building blocks for smart devices developed by all industries [6]. The resulting fibrous architectures can be employed as three-dimensional porous scaffolds for hard [3,7] or soft [8] tissues, sacrificial templates [9], photocatalytic materials [10,11], transparent flexible electrodes [12], semiconducting elements [13] or light sources [14].

Additionally, bioactive glasses constitute an emerging research field for the development of bone porous scaffolds, given that most of the used compositions are included in the category of melt-derived bioactive silicate glasses [1]. In this case, the relationship between porosity and mechanical strength has been systematically investigated and modeled in order to contribute to the rational design of devices with custom-made properties [15]. Equally important is their reactive surface, which triggers the ability of attaching to living hard tissue in a physiological environment by promoting the formation of strong chemical bonds [16]. The sol-gel method has been proposed as a valuable alternative route for producing glasses with an improved bioactivity, due to the adapted composition and microstructural features, as well as the low synthesis temperature [17,18].

The crystallization process enhances the mechanical properties of glasses, leading at the same time to a decrease in the bioactivity level; the resulting glass-ceramics consist of crystalline phases embedded in a glassy matrix [1]. The thermal processing must be conducted in such a way that the nucleation and growth of crystalline domains provide a balance between multiple aspects; however, the final three-dimensional porous structure should allow for cell proliferation, vascularization and nutrient diffusion [19]. Such materials, which are of an intrinsically composite character, can be shaped either as scaffolds [20,21] or as coatings [22,23].

Using this idea of combining phases of different natures in order to reach superior performances, hydroxyapatite synthesized from eggshell waste was composited with PCL [24], and PCL microfibers containing dispersed alumina and selenite-doped carbonated hydroxyapatite were obtained by electrospinning [4], both systems showing beneficial influences on the tested cell cultures. Moreover, PCL and bioactive glass nanoparticles were integrated through different processing techniques in several types of complex materials [25,26,27], which revealed excellent mechanical properties, an outstanding apatite-forming ability and an enhanced cell proliferation/differentiation. Gomez-Cerezo et al. [28] even demonstrated, both in vitro and in vivo conditions, the suitability of macroporous scaffolds made of mesoporous bioactive glass and PCL in a scenario of osteoporosis. Such composites were also tested in the form of coatings on metallic supports, with the aim of reducing corrosion and facilitating mineralization [29]. Regarding the replacement of glasses with glass-ceramics, the territory has not been explored much, with detailed studies being required in order to formulate the guiding principles; Naghizadeh et al. [30] fabricated a three-dimensional porous scaffold using PCL and bioactive silicate-based glass-ceramic prepared from rice husk ash, the material being useful as a bone substitute in non-load bearing sites.

Our research group also had concerns in relation to the stimulation of bone regeneration, proposing two families of composite fibrous scaffolds synthesized by electrospinning. In the first case, PCL was combined with zinc oxide, titanium dioxide or hydroxyapatite in order to achieve extra bioactivity or antibacterial activity [7], while in the second one PCL was loaded with hydroxyapatite or/and barium titanate, targeting biocompatible, bioresorbable and bioactive features combined with external stimulation capabilities [3].

In this context, we report on the integration of glass-ceramic nanopowders in electrospun polymeric fibers, the emphasis being on the effects of Mg^2+^ and Sr^2+^ cations from the powder composition, as well as on the repercussions of inorganic particles being embedded within polycaprolactone one-dimensional structures. The novelty of such an approach is based on the oxide powder composition, from which new mineralogical characteristics derive, but also on the combination, made for the first time, of the mentioned organic and inorganic components, our aim being to propose multifunctional systems with enhanced effects on tissue regeneration.

## 2. Materials and Methods

### 2.1. Inorganic Powders Synthesis

Two inorganic powders were synthesized following a sol-gel route, with the aim of achieving pure, homogenous and nanometric particles that can be easily loaded on a fibrous polymeric scaffold. The established compositions are displayed in Table 1, where the difference is clearly highlighted: one contains magnesium and the other strontium. These two cations (Mg^2+^ and Sr^2+^) were selected based on their beneficial contributions in relation to mechanical resistance, bioactivity and cellular stimulation [21,31,32]. All other oxides have important roles as network formers or modifiers, mineralization/crystallization agents or antibacterial activity providers [33]. Moreover, the SiO_2_ proportion is placed at the upper limit when it comes to the bioactivity mechanism, a fact that has been correlated with the behavior of the employed bioresorbable polymer and its degradation time [2]. Generally, partially crystallized glasses, namely glass-ceramics, are targeted in this study, with the purpose of reinforcing the resistance structure, even though the bioactivity level will be compromised to a certain extent [1].

In relation to the experimental procedure, a clear and homogenous precursor solution containing all described cations was achieved, and was subsequently subjected to the well-known stages of gelation, aging, drying and calcination; the latter was performed at 600 °C for 2 h, resulting in the designed inorganic powder.

### 2.2. Composite Scaffolds Fabrication

To obtain composite fibers arranged in a three-dimensional porous scaffold, electrospinning solutions were prepared according to the data listed in Table 2. First, a suspension of oxide powder in a solvent mixture (chloroform—CF and dimethylformamide—DMF) was obtained through ultrasonication for 5 min, after which polycaprolactone (PCL, *M_W_* = 80,000 g/mol) was added and magnetically stirred for 24 h in order to break the particle aggregates from the powder on the one hand, and ensure the total dissolution of the polymer on the other. The final solution was loaded in a plastic syringe with a blunt-tip stainless steel needle with a 0.8 mm inner diameter and was fed with the help of a syringe pump at a rate of 3 mL/h, under an applied high voltage of 15 kV; the static planar collector was placed at a distance of 20 cm from the spinneret tip, while the environmental parameters were 24 °C temperature and 30% relative humidity. The electrospinning process was carried out until a consistent mat of randomly distributed fibers was deposited on the glass slides attached to the collector. The resulting samples were named similarly to the solutions from which they were electrospun (Table 2).

### 2.3. Materials Characterization

The characterization was performed from multiple perspectives, as follows: complex thermal analysis (Shimadzu DTG-60 equipment, Shimadzu Corporation, Kyoto, Japan), scanning electron microscopy coupled with energy-dispersive X-ray spectroscopy (SEM+EDX, FEI Quanta Inspect F electron microscope, FEI Company, Hillsboro, OR, USA), Fourier-transform infrared spectroscopy (FTIR, Thermo Scientific Nicolet iS50 spectrophotometer, Thermo Fisher Scientific, Waltham, MA, USA), X-ray diffraction (XRD, Shimadzu XRD 6000 diffractometer, Shimadzu Corporation, Kyoto, Japan) and simulated body fluid (SBF) immersion (Kokubo solution [34], pH = 7.3, 37 °C, 4 weeks).

### 2.4. Cellular Evaluation

A human fetal osteoblast cell line (hFOB 1.19, ATCC, Manassas, VA, USA) was cultivated in MEM α (A1049001, Gibco, Waltham, MA, USA) supplemented with 10% Fetal Bovine Serum (FBS, F7524, Sigma, St. Louis, MO, USA) and 1% Penicillin-Streptomycin (P4333, Sigma) and seeded at different densities on PCL-Mg-1, PCL-Mg-5, PCL-Sr-1 and PCL-Sr-5 scaffolds. The cells were statically seeded and left to adhere for 30 min. Consequently, the cell culture medium was added, and the samples were incubated at 37 °C in 5% CO_2_ for 24 or 48 h. The cellular viability was assessed with a LIVE/DEAD Viability/Cytotoxicity Kit for mammalian cells (L3224, Life Technologies, Waltham, MA, USA). The cells were seeded on scaffolds placed in 48-well plates at a density of 1 × 10^5^ cells/well. After 24 h in culture, the samples were rinsed with 1× Phosphate Buffered Saline (PBS, P3813, Sigma) to remove serum esterase activity and were incubated for 30 min at room temperature with 2 µM Calcein-AM and 4 µM Ethidium Homodimer-1. After incubation, 1× PBS was added, and the viability was evaluated with a Zeiss LSM 880 confocal microscope (Zeiss, Oberkochen, Germany) using 488 and 514 nm lasers and processing with ZEN 2.3 software (Zeiss, Oberkochen, Germany).

The confocal fluorescence microscopy with CellTracker Red CMTPX (C34552, Molecular Probes, Eugene, OR, USA) was performed after 24 h in culture. The CellTracker fluorescent samples (1:1000 dilution) were incubated for 30 min at 37 °C and imaged with a 514 nm laser, employing the same confocal microscope.

In order to assess the cellular adhesion, the actin filaments were stained with Fluorescein Phalloidin (F432, Thermo Fisher Scientific, Waltham, MA, USA). The samples’ fixation was performed with 3.7% formaldehyde in PBS solution (P733.1, Roth, Dautphetal, Germany) for 30 min at room temperature. After the PBS wash, the permeabilization was performed with 0.3% Tween 20 in PBS solution for 1 h. The blocking of non-specific binding sites was achieved with 3% Bovine Serum Albumin (BSA) dissolved in 0.3% Tween 20 in PBS solution, followed by staining with 4 µg/mL Fluorescein Phalloidin in the blocking solution and incubation for 1 h at room temperature. PBS wash was performed prior to the application of ProLong Gold Antifade Mountant with DAPI (P36931, Life Technologies, Waltham, MA, USA). Fluorescein Phalloidin was stimulated at 488 nm and evaluated at 500–631 nm, using a 52.6 pinhole (1.68 Airy Units); DAPI was excited at 405 nm and evaluated at 409–451 nm, using a 52.6 pinhole (2.21 Airy Units).

## 3. Results and Discussion

### 3.1. Inorganic Powders Characterization

**Figure 1:**
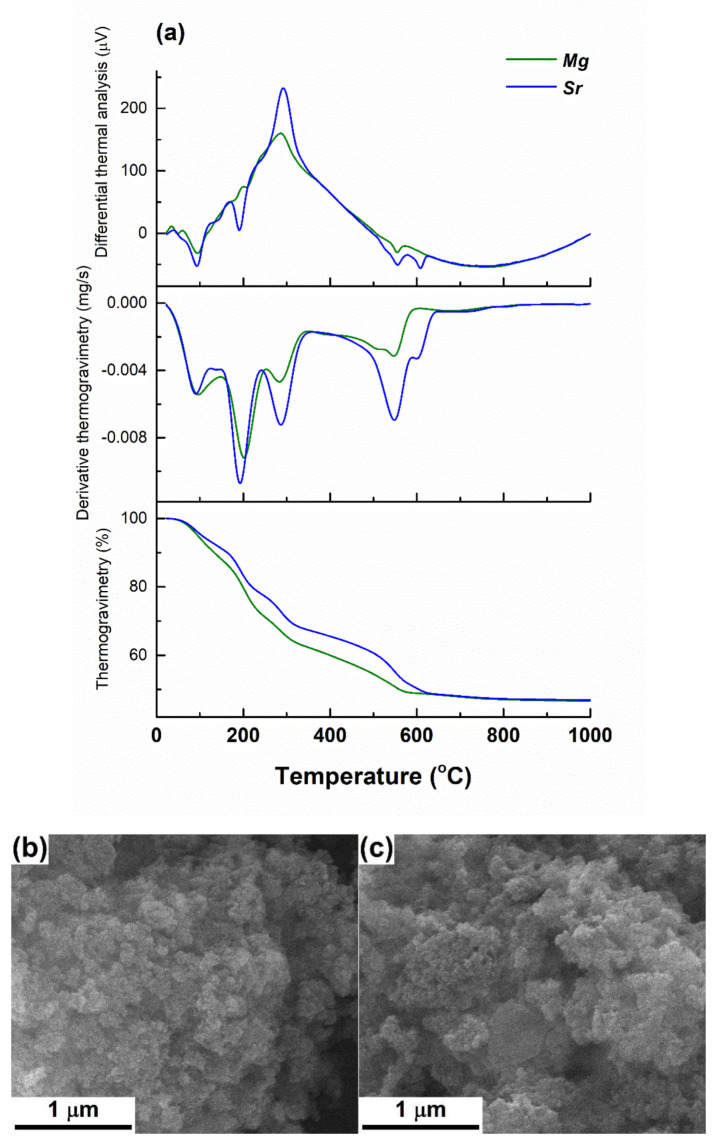

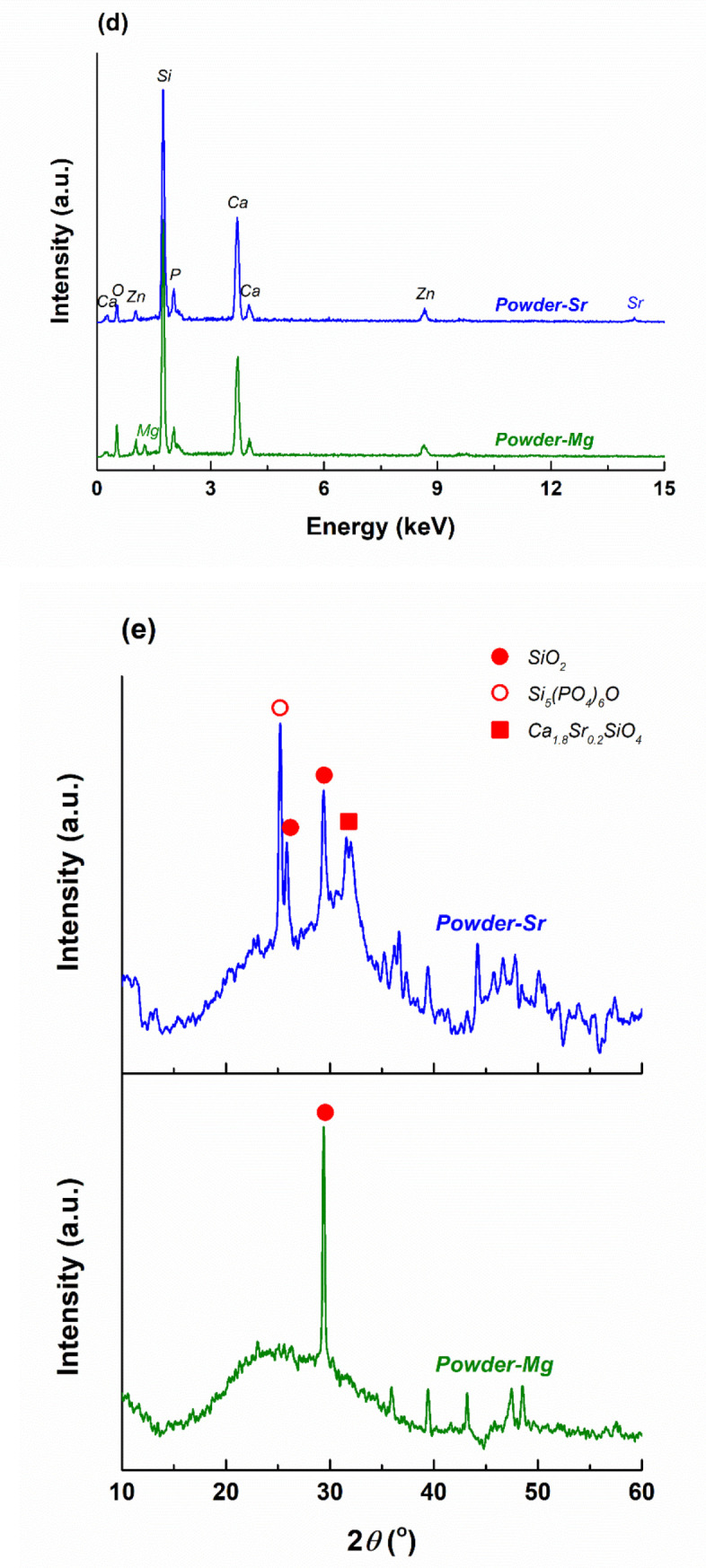
(**a**) Thermal analyses on the dried gels, SEM images of: (**b**) Powder-Mg and (**c**) Powder-Sr, (**d**) EDX spectra and (**e**) XRD patterns of the calcined powders.

First, the inorganic powders were characterized in order to highlight their morphology, composition and structure. Before this stage, thermal analyses performed on the dried gels were necessary to determine the appropriate calcining temperatures; thus, Figure 1a presents the recorded curves giving gravimetric and thermal information about the two compositions. As can be seen, the behavior is quite similar, most of the volatile or degradable components being removed up to 600 °C, even though the slope shows some changes from one sample to the other. Moreover, the presence of magnesium nitrate or strontium nitrate generates a different succession of weight losses or endothermic thermal effects around 600 °C.

Figure 1b,c exhibits the aspect of the calcined powders, which consists of spherical particles with an average diameter of approximately 25 nm for Powder-Mg and 30 nm for Powder-Sr; their size distribution is extremely narrow, being gathered as micrometric aggregates. The process of breaking these aggregates is mandatory but extremely difficult due to the high surface energy, which triggers such an assembly so as to minimize the overall energy.

The associated EDX spectra (Figure 1d) confirm the existence in the calcined powders of all elements introduced in the precursor solutions, with the ratios between the identified lines being compatible with the selected concentrations.

The most suggestive investigation is displayed in Figure 1e, giving valuable indications in terms of the crystalline character and structure type. Thus, both XRD patterns evidence the occurrence of a glassy phase through the large halo, from which additional diffraction peaks emerge, as a proof of partial crystallization within the primary amorphous matrix. In the case of Powder-Mg, the main crystalline phase was identified as monoclinic SiO_2_ (ICDD 00-083-1833), while in the case of Powder-Sr several crystalline compounds were assigned: monoclinic or hexagonal SiO_2_ (ICDD 00-083-1833 and ICDD 00-081-1665), rhombohedral Si_5_(PO_4_)_6_O (ICDD 00-081-1592) and orthorhombic Ca_1.8_Sr_0.2_SiO_4_ (ICDD 00-077-1621). The latter constitutes a proof of strontium partial substitution on calcium sites from the Ca_2_SiO_4_ network, based on a small difference between their ionic radii (1.00 Å versus 1.18 Å). It is also obvious that the composition including strontium is more prone to crystallization, this element being well-known for having such a behavior [35].

In conclusion, the two oxide powders that will be integrated in the polymeric fibers are in the form of partially crystallized materials, which are suitable for various applications in the medical field, since they contain a large amount of vitreous phase, as well as certain beneficial crystalline phases. According to the scientific literature, the amorphous matrix increases the bioactivity through the labile structure [1,16,36], while the crystalline compounds seem to express their influence at the level of mechanical properties, cellular proliferation/differentiation favoring or osteogenesis stimulation [37,38,39].

### 3.2. Composite Scaffolds Characterization

**Figure 2:**
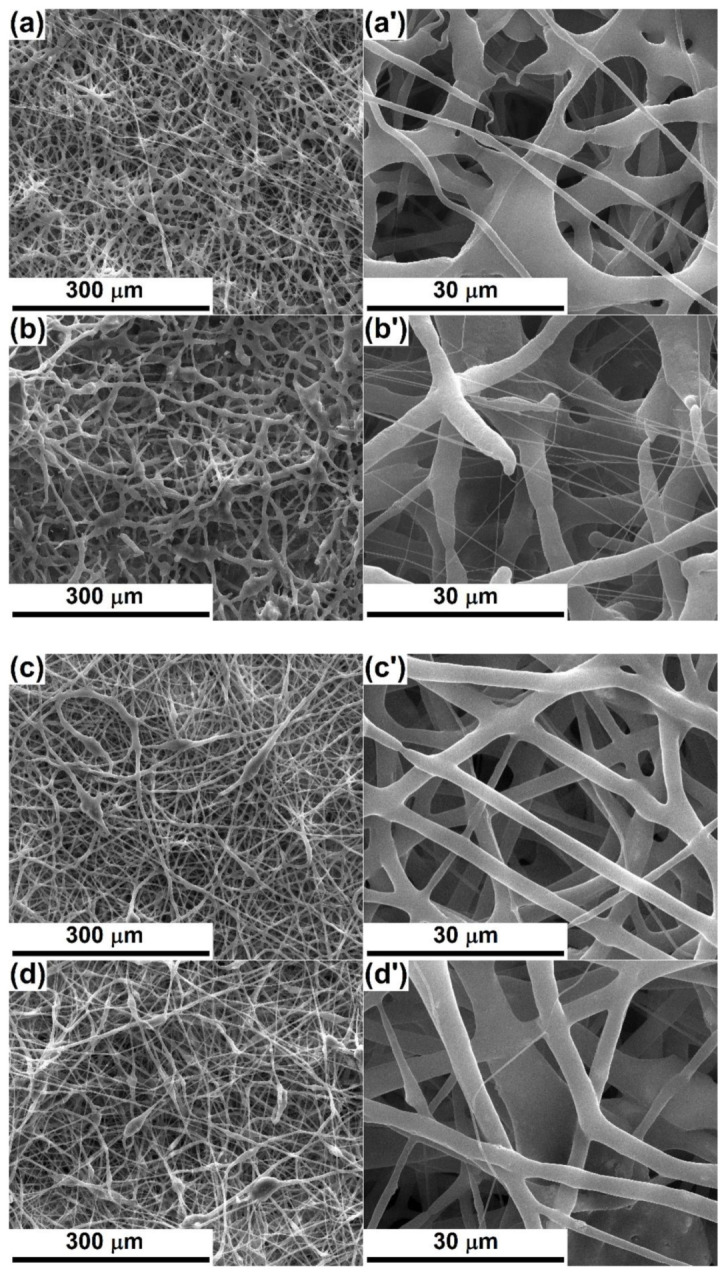
SEM images, based on secondary electrons at different magnifications, of: (**a**,**a’**) PCL-Mg-1, (**b**,**b’**) PCL-Mg-5, (**c**,**c’**) PCL-Sr-1 and (**d**,**d’**) PCL-Sr-5 scaffolds.

Figure 2 shows SEM images based on the topographic contrast for all four composite scaffolds obtained by electrospinning. In order to understand the effects of the oxide powders on the resulting morphology, an evaluation of bare polymeric fibers was needed. According to [3,7], where similar processing parameters were applied for the fabrication of PCL fibers, the one-dimensional entities are non-woven, arranged randomly in several tens of overlapping layers and have diameters of around 3 μm; their length cannot be estimated, but instead a great electrostatic connection/sticking tendency appears sometimes. Moreover, the fiber surface is quite smooth, the diameter relatively constant along the entire length and the general appearance curly/winding, suggesting a high flexibility of the whole scaffold; very rarely can fibers with a much smaller diameter (800 nm) or areas with electrospinning defects be detected.

The addition of Powder-Mg at a concentration of 1% or 5% leads to a change in the overall appearance, the fibers losing their cylindrical shape or fragmenting from place to place (Figure 2a,b). The fiber diameter is no longer constant over a long distance, while the interconnection degree increases, which converts the scaffolds into strengthened networks due to many connectivity points. From a dimensional point of view, there are at least two categories of fibers in the case of PLC-Mg-1 (Figure 2a,a’), namely thicker (4 μm) and thinner (1 μm) ones. Increasing the powder proportion from 1 to 5% (Figure 2b,b’) causes the thin fibers to become even thinner (200 nm) and their proportion to increase, which suggests a major modification in the mechanism of the electrospinning process with the addition of a larger amount of inorganic powder in the precursor solution.

In the SEM images of PCL-Sr-1 and PCL-Sr-5 (Figure 2c,d), it is evident that the destruction of the initial homogeneous morphology of the bare PCL scaffold occurs to a lesser extent than in the previous case, keeping a relatively constant diameter over long length, with the fibers’ union manifesting only occasionally. The significant difference is represented by the emergence of “beads on string”-type structures, equivalent to a local thickening of the fiber, most likely due to the localization of particle aggregates from powder. While the average fiber diameter remains around 4 μm, the “beads” can even reach 20 μm (Figure 2c,c’). Raising the powder concentration from 1 to 5% (Figure 2d,d’) determines the growth of the number of “beads” but also a general thinning of the fibers (3 μm) and the appearance of very thin fibers (500 nm).

**Figure 3:**
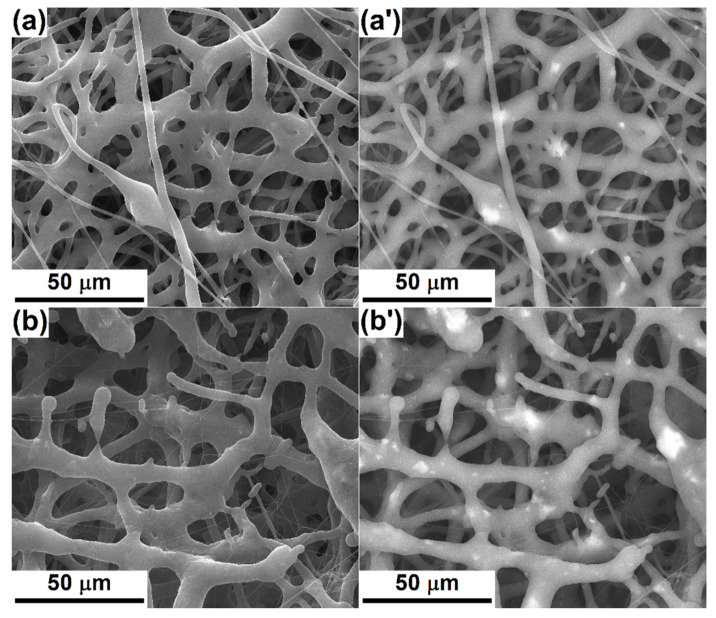

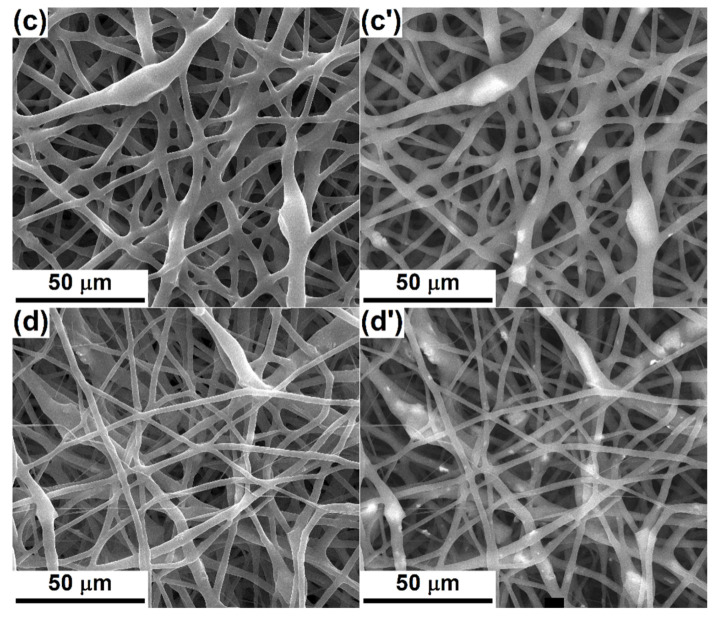
SEM images, based on secondary electrons (left) or backscattered electrons (right), of: (**a**,**a’**) PCL-Mg-1, (**b**,**b’**) PCL-Mg-5, (**c**,**c’**) PCL-Sr-1 and (**d**,**d’**) PCL-Sr-5 scaffolds.

In order to assess the way in which the oxide powders are distributed within the scaffolds, SEM images based on atomic number contrasts were employed, knowing that they provide compositional information about a material. As a result, Figure 3 comparatively shows SEM images acquired using secondary (Figure 3a–d) or backscattered (Figure 3a’–d’) electrons, selecting the same area of the specimen. The occurrence of brighter regions in the second category of images is generated by the local existence of heavier elements, in this case: Si, P, Ca, Zn and Mg/Sr. The powders’ distribution is relatively homogeneous on the investigated areas, being individualized as micrometric aggregates embedded in the fibers’ volume; the loading effect takes place mainly at those points where the fiber diameter is thickened or where the fibers intersect (Figure 3a’). As expected, the number of bright spots increases in the case of PCL-Mg-5 (Figure 3b’), which confirms the stability of the precursor solution, even in the case of a higher powder proportion, but also a higher loading degree of the final material with inorganic powder. With regard to PCL-Sr-1 (Figure 3c’) and PCL-Sr-5 (Figure 3d’), the localization of the particle aggregates inside the “beads” is validated, as is the increase in the powder concentration from the first to the second sample, as designed.

**Figure 4:**
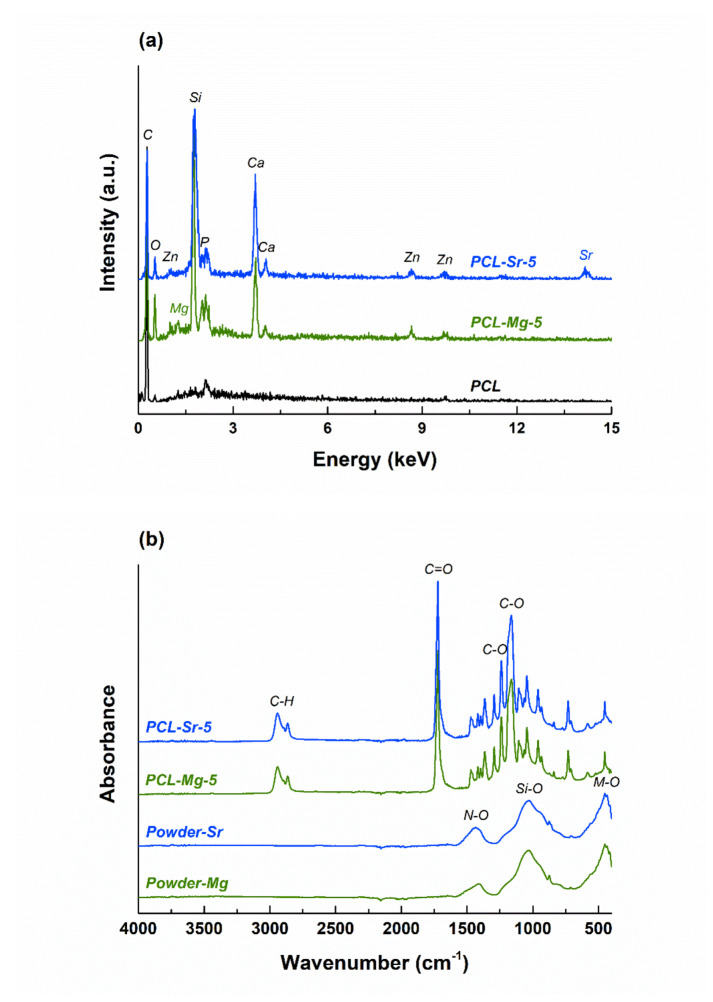
(**a**) EDX of PCL, PCL-Mg-5 and PCL-Sr-5 scaffolds and (**b**) FTIR spectra of Powder-Mg, Powder-Sr, PCL-Mg-5 and PCL-Sr-5 scaffolds.

The loading process was also highlighted through the EDX investigation; thus, the EDX spectra of the scaffolds containing 5% inorganic powder are integrated in Figure 4a, together with the EDX spectrum of the bare PCL sample. If in the case of PCL the main elements of the polymer (C and O) are visible, additional elements specific to the oxide powders’ composition can be found in the other two spectra, namely Si, P, Ca, Zn, Mg/Sr and O.

Figure 4b displays the FTIR spectra of the same materials as earlier, but comparatively to the corresponding spectra of the embedded inorganic powders. For Powder-Mg and Powder-Sr, large vibrational bands can be observed and attributed to typical bonds, mainly Si‒O and M‒O, where *M* = Ca, Zn and Mg/Sr [40]. In relation to the scaffolds, multiple narrow and intense peaks are shaping up as proofs of PCL bonds (C‒H, C=O and C‒O) [41]; below 1500 cm^−1^, they emerge in the form of spikes arising on top of the mentioned large band. The presence of N‒O bonds can be explained by residual nitrate groups [33].

### 3.3. Biological Evaluation

**Figure 5:**
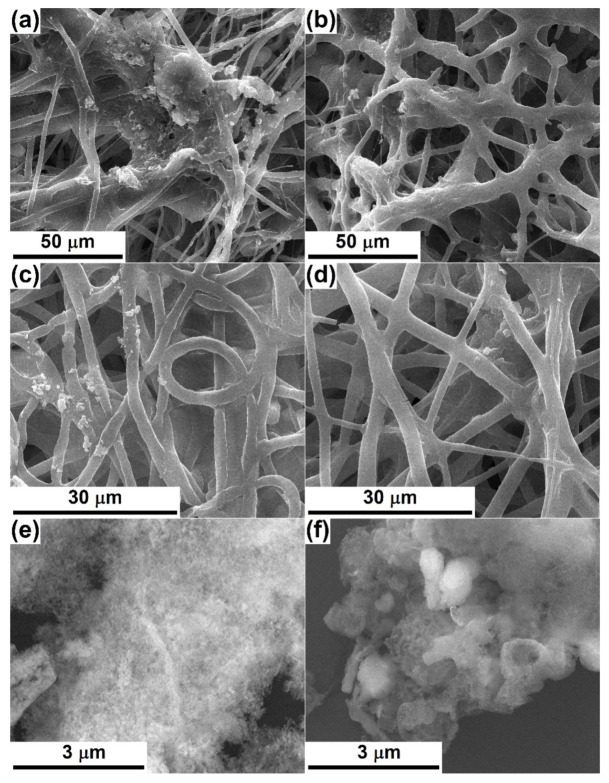
SEM images, based on secondary electrons after four weeks of immersion in SBF, of: (**a**) PCL-Mg-1, (**b**) PCL-Mg-5, (**c**) PCL-Sr-1 and (**d**) PCL-Sr-5 scaffolds, and (**e**) Mg-containing and (**f**) Sr-containing compositions.

After four weeks of immersion in SBF, the samples present light signs of degradation or mineralization. At the level of the polymer, the most important changes can be described as follows: fibers thinning (Figure 5a), thin fibers collapsing and being embedded in thick fibers (Figure 5b), fibers flattening slightly, where fibers lose their rounded cylindrical appearance and acquire a flat shape, probably on the sides through contact with other surfaces (Figure 5c), or fiber interruption/cracking, where fibers are completely sectioned or only cracked on their surface (Figure 5d). If the period of immersion would have been prolonged, they probably would have been gradually destroyed and transformed into compounds that could be safely removed by the body excretory system. The particle aggregates trigger a shallow bioactivity, which is proven by the local formation of small and loose apatite entities on favorable areas (Figure 5a,c). Due to the reduced amount of oxide powder, but also to the fact that this glass-ceramic phase is embedded in the polymer (the reactive surface is not available for reactions with SBF), the effect is delayed, probably needing a higher loading degree or more immersion time in SBF. Since the powder represents a discontinuous phase, it should ensure the availability of active centers, from where mineralization begins. The compatibility of the resorption and mineralization rates is an essential aspect, and great attention will be invested in the future in order to elucidate such a personalized approach. In order to clearly demonstrate the bioactivity of both employed compositions, they were processed in the form of films to ensure an extended and exposed surface for reactions with SBF during the four weeks of immersion. These surfaces exhibit, from place to place, apatitic formations having a loose globular nanostructured morphology, characteristic of the phase emerging following a mineralization process; sometimes, raspberry-like structures are visible, frequently reported in the scientific literature as being a mineralization product [17].

**Figure 6:**
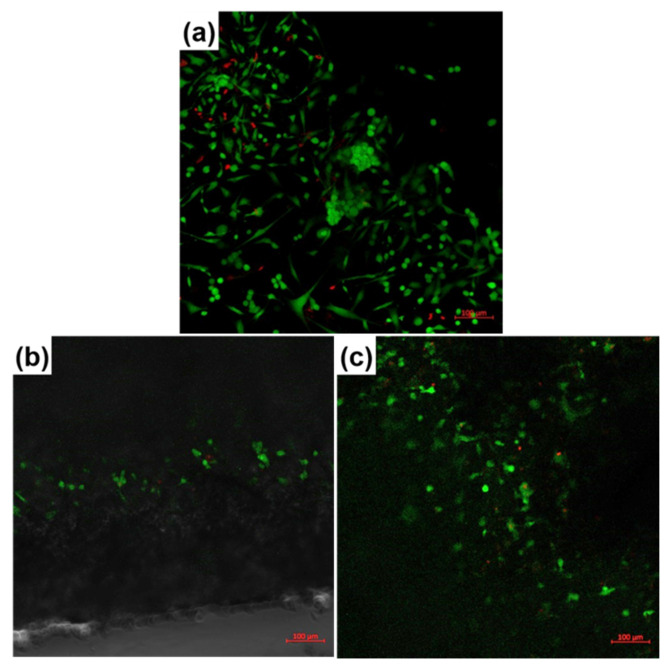

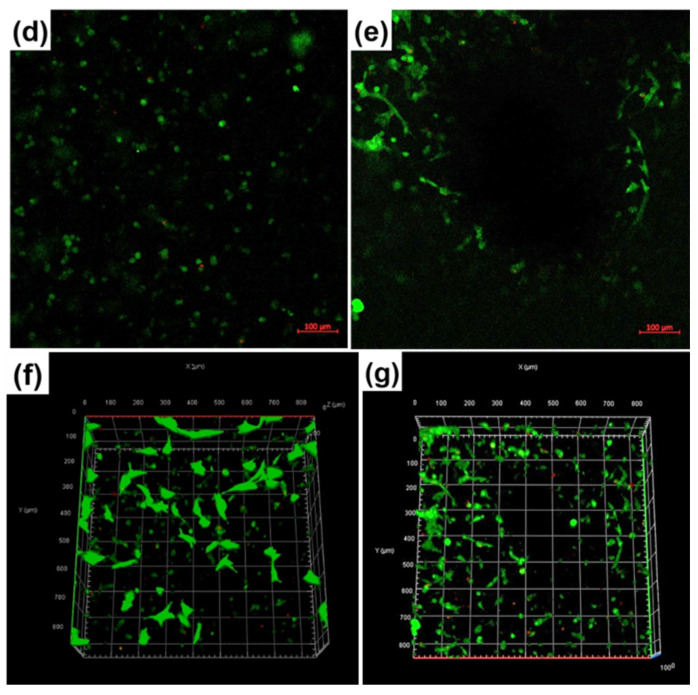
(**a**) Fluorescence microscopy images recorded on fetal osteoblasts cultured for 24 h of: (**a**) Control, (**b**) PCL-Mg-1, (**c**,**f**) PCL-Mg-5, (**d**) PCL-Sr-1 and (**e**,**g**) PCL-Sr-5 scaffolds.

The impact of the fabricated composite samples on human fetal osteoblasts was estimated through the medium of the LIVE/DEAD assay and fluorescence microscopy after 24 h in culture, the results being revealed in Figure 6. The LIVE/DEAD assay supposes the fluorescence marking of living cells with Calcein-AM and dead cell nuclei with Ethidium Homodimer-1, which makes the viable cells green and the nuclei of dead cells red. The cells’ visualization was difficult due to the specific morphology of the fibrous scaffolds, with superposed layers of fibers among which the cells can easily penetrate; therefore, the cells adhering to the material can be detected successively at increasing depths. This means that a *Z*-stacking approach was conducted, allowing for the acquisition of multiple images at different focal distances and the subsequent integration in a final image with a greater depth of field. The results validated cellular adhesion especially in the material volume, to the detriment of its surface. Almost all identified cells are viable, and sometimes they seem to be connected in small networks, probably following the fibers’ direction. For both inorganic powders, a higher cellular adhesion and proliferation can be noticed at a 5% concentration. A better evidence of the scaffolds’ adequate biocompatibility is provided by Figure 6f,g, in which three-dimensional reconstructions of the investigated areas are shown; these confirm the cells’ migration in the entire volume, as well as a normal cellular metabolism.

**Figure 7:**
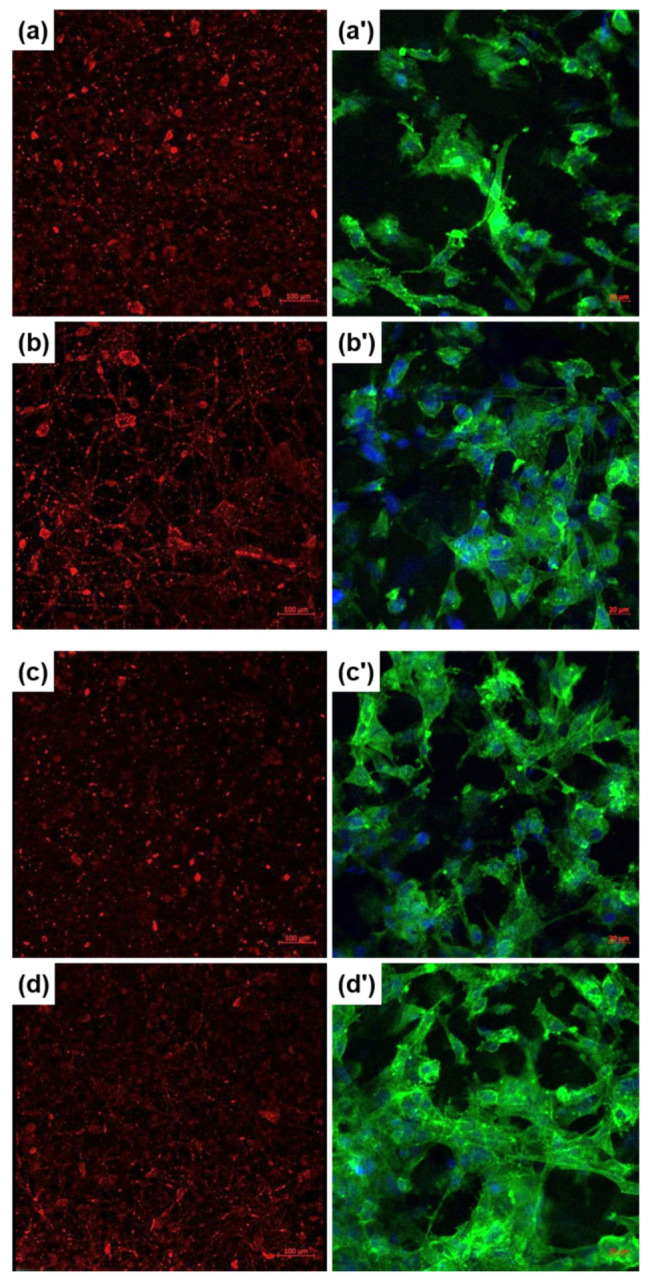
Fluorescence microscopy images recorded on fetal osteoblasts cultured for 24/48 h of: (**a**,**a’**) PCL-Mg-1, (**b**,**b’**) PCL-Mg-5, (**c**,**c’**) PCL-Sr-1 and (**d**,**d’**) PCL-Sr-5 scaffolds, achieved with: (**a**–**d**) CellTracker Red CMTPX or (**a’**–**d’**) Fluorescein Phalloidin with DAPI.

Additionally, CellTracker Red CMTPX is a fluorescent dye that is suitable for monitoring cells’ location and movement through the deep red emission. As a consequence, the homogenous coloring of the images obtained with the help of this fluorophore emphasizes a fairly even cellular distribution on the investigated areas (Figure 7a–d). Otherwise, Calcein-AM, employed in the first biological assay, as well as CellTracker Red CMTPX, involved in the second cellular evaluation, require an enzymatic activity in order to be converted into fluorescent molecules, a fact that can be readily correlated with the development of an intracellular metabolism in a standard regime.

Eventually, Fluorescein Phalloidin was used to selectively label the actin filaments with Phalloidin, and then Fluorescein provided their high-contrast visualization through the green fluorescence. Moreover, DAPI blue fluorescent dye ensured a nuclear counterstaining. Thus, the actin filaments stained with Fluorescein Phalloidin reveal the cellular adhesion at the composite fibrous scaffolds, highlighted through a flattened morphology with multiple extensions, as well as through the formation of cellular networks evidenced by the cells’ proximity and the convergence of actin filaments at the cells’ contact areas (Figure 7a’–d’).

## 4. Conclusions

Composite scaffolds were fabricated by successfully combining the sol-gel method for the synthesis of the inorganic powders with the electrospinning technique for the generation of the fibrous aspect. Two types of oxide powders were used, one containing magnesium and the other strontium, their nanometric size and glass-ceramic nature being validated through specific investigations. Polycaprolactone was selected as a bioresorbable matrix, in which the powders were embedded in concentrations of 1% or 5%. The morphological characteristics of the loaded scaffolds varied as a function of the powder composition and proportion, these two parameters modifying the shape, dimension and interconnection of fibers. A slight fiber degradation and mineralization with apatite phases were highlighted after immersion in the simulated biological environment for four weeks. The biological assessment was performed on osteoblast cells and indicated a good biocompatibility of all the obtained materials. Our next work will be focused on more extended investigations on the bioresorbability and bioactivity correlation from a rate point of view.

## Figures and Tables

**Table 1:** Composition of the synthesized oxide powders and the corresponding reagents.

Oxide	Concentration (mol%)		Precursor
	Powder-Mg	Powder-Sr	
SiO_2_	60	60	Si(OC_2_H_5_)_4_ (TEOS) (Aldrich, St. Louis, MO, USA)
P_2_O_5_	5	5	PO(OC_2_H_5_)_3_ (TEP) (Merck, St. Louis, MO, USA)
CaO	25	25	Ca(NO_3_)_2_·4H_2_O (Merck, St. Louis, MO, USA)
ZnO	5	5	Zn(NO_3_)_2_·6H_2_O (Sigma-Aldrich, St. Louis, MO, USA)
MgO	5	-	Mg(NO_3_)_2_·6H_2_O (Merck, St. Louis, MO, USA)
SrO	-	5	Sr(NO_3_)_2_ (Sigma-Aldrich, St. Louis, MO, USA)

**Table 2:** Composition of the electrospinning solutions.

Solution	Components			
	PCL (g)	Solvent Mixture (CF:DMF = 4:1) (mL)	Powder-Mg (g)	Powder-Sr (g)
PCL	1.6	10	-	-
PCL-Mg-1	1.6	10	0.1	-
PCL-Mg-5	1.6	10	0.5	-
PCL-Sr-1	1.6	10	-	0.1
PCL-Sr-5	1.6	10	-	0.5
